# Multifunctional Electrospun Phase Change Material Mats for Solar–Thermal Energy Storage and Photothermal Conversion

**DOI:** 10.1002/smsc.202500600

**Published:** 2026-04-17

**Authors:** Hossein Baniasadi, Sedigheh Borandeh, Ziba Fathi, Roozbeh Abidnejad, Pedro E. S. Silva, Lauri Välinen, Jaana Vapaavuori, Eero Kontturi, Jukka Niskanen

**Affiliations:** ^1^ School of Chemical Engineering Department of Chemical and Metallurgical Engineering Aalto University Espoo Finland; ^2^ School of Chemical Engineering Department of Bioproducts and Biosystems Aalto University Espoo Finland; ^3^ School of Chemical Engineering Department of Chemistry and Materials Science Aalto University Espoo Finland

**Keywords:** electrospun mats, multifunctional materials, phase change materials, photothermal conversion, thermal energy storage

## Abstract

Efficient solar–thermal energy conversion and storage demand materials that combine high latent heat with multifunctional performance were observed. Here, we report electrospun polyester‐based phase change material (PCM) mats capable of simultaneous thermal energy storage and photothermal conversion. Among a series of PCMs, PPCM1218 (from 1,12‐dodecanediol and octadecanedioic acid) showed the highest latent heat (157 J/g, melting point 90.6°C) and was incorporated into flexible, bead‐free mats via blending with polyamide 11 to prevent leakage. Mats with high PCM loading retained up to 132 J/g enthalpy while maintaining mechanical integrity. Functionalization with biochar, graphene, or polypyrrole (PPy) enhanced stiffness, electrical, and thermal conductivity, with PPy‐coated mats achieving 0.51 W/m·K and 18.5 S/m. Under 1.5 sun irradiation, surfaces exceeded 90°C, fully melting the PCM and demonstrating stable energy storage and release over 100 thermal and photothermal cycles. This approach offers a versatile platform for multifunctional electrospun mats suitable for high‐temperature energy storage, solar‐driven water purification, and thermal management applications.

## Graphical abstract

Electrospun polyester‐based phase change mats were functionalized with biochar, graphene, or polypyrrole to enhance photothermal performance. The composites maintain fibrous morphology while achieving tunable mechanical, thermal, and electrical properties. Polypyrrole‐coated mats show significantly improved light‐to‐heat conversion, demonstrating their potential for efficient solar‐driven thermal energy harvesting and storage applications.

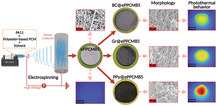

## 1 Introduction

The global demand for energy‐efficient technologies and sustainable thermal management has intensified the search for advanced materials capable of storing, regulating, and harvesting thermal energy [1, 2]. Among these, phase change materials (PCMs) are particularly attractive due to their ability to store and release large amounts of latent heat during phase transitions [3, 4, 5]. This capability underpins applications in energy‐saving building materials [6], packaging [7], smart textiles [8], biomedical textiles [9], personal thermal regulation [10], and wearable electronics [11].

Incorporating PCMs into fiber‐based matrices enables flexible, lightweight systems for passive temperature regulation and efficient heat management. Techniques, such as melt spinning [12], wet spinning [13], and electrospinning [14], have been widely used, with electrospinning favored for producing nanofibers with high specific surface area. Functional additives—including carbon‐based nanomaterials (graphene derivatives) and conductive polymers (polypyrrole (PPy), polyaniline)—further enhance photothermal conversion and electrical conductivity, enabling responsive thermal management. Representative studies include stretchable fibers embedding PCM microcapsules in carbon‐reinforced polyurethane [15], electrospun polyamide/decanoic acid fibers modified with PPy [16], core–sheath fibers of octadecane and titanium carbide achieving high latent heat and photothermal efficiency [17], and paraffin wax encapsulated in polyacrylonitrile sheaths with Cs_0_._32_WO_3_ nanoparticles showing high encapsulation efficiency and long‐term stability [18].

Despite these advances, polyester‐based PCMs (PPCMs) have received comparatively less attention, even though they offer tunable thermal properties, mechanical stability, and excellent processability for fiber‐based applications [19]. PCMs can be synthesized via ring‐opening polymerization, polycondensation, or microbial fermentation. Several studies report solid–solid PPCMs with enhanced form stability, high latent heat (≈ 120–139 J/g), and excellent thermal reliability. For example, polyethylene glycol (PEG)‐based PPCMs prepared via solvent‐free crosslinking or esterification, as well as ionic crosslinked PEG polyesters, maintain performance after thermal cycling and enable recyclability under high temperature and pressure [20, 21, 22].

While solid–solid PPCMs often require complex covalent or ionic crosslinking, solid–liquid PPCMs offer a simpler, scalable route. They can be prepared via direct polycondensation of diols and diacids, allowing tunable melting temperatures, crystallinity, and latent heat through monomer chain‐length selection [23, 24, 25]. However, like other solid–liquid PCMs, they can suffer from leakage during melting, which compromises thermal reliability and flexible substrate compatibility [26, 27]. Effective strategies to stabilize the molten phase within fiber matrices are therefore critical for safe, durable, and high‐performance smart textiles.

Polyamides (PAs), or nylons, are widely used in textiles and engineering due to their mechanical strength, chemical resistance, and thermal stability [28, 29]. Their strong hydrogen bonding and excellent spinnability make them well suited for electrospun nanofibrous structures [30, 31]. Among them, PA11, a fully biobased aliphatic PA, has shown promise for advanced fiber applications [32], and in our previous work, it demonstrated excellent electrospinnability while effectively stabilizing a solid–liquid decanoic acid PCM [16]. Its polar amide groups form hydrogen bonds with ester carbonyl (C=O) and residual hydroxyl (–OH) groups of PPCMs, enhancing dispersion, interfacial adhesion, and durability within composite fibers, making it an ideal matrix for high‐performance PPCM‐based smart textiles.

Herein, PPCMs were synthesized and designated PPCM1010, PPCM1212, and PPCM1218, reflecting the chain lengths of the diols and diacids used. PPCM1218 was subsequently blended with PA11 to produce electrospun mats (ePPCM85), which were further functionalized with biochar (BC@ePPCM85), graphene (Gr@ePPCM85), or surface‐polymerized PPy (PPy@ePPCM85). These engineered mats exhibit high latent heat (up to 132 J/g), excellent shape stability, rapid solar‐to‐thermal energy conversion, and robust durability under repeated thermal and photothermal cycling. The combination of energy storage and photothermal responsiveness positions these mats as promising candidates for next‐generation smart textiles, flexible sunlight collectors, and high‐temperature thermal management applications.

## 2 Results and Discussion

### 2.1 Polyester Phase Change Materials Characterization

**FIGURE 1:**
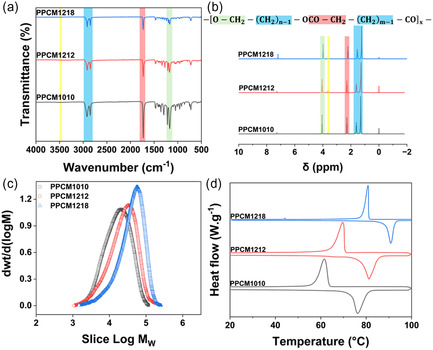
Structural and thermal characterization of the synthesized PPCM. (a) FTIR spectrum, (b) ^1^H NMR spectrum, (c) molecular weight distribution, and (d) DSC thermograms. *n* = number of CH_2_ units in the diol, *m* = number of methylene (–CH_2_–) units in the diacid, and *x* = number of repeating units. All measurements were conducted on representative samples, and the data shown are representative of the obtained results. DSC = Differential scanning calorimetry; FTIR = Fourier‐transform infrared; ^1^H NMR = proton nuclear magnetic resonance; PPCM = polyester‐based phase change material.

The successful synthesis of the PPCMs, including PPCM1010, PPCM1212, and PPCM1218, was confirmed through complementary Fourier‐transform infrared (FTIR) and proton nuclear magnetic resonance (^1^H NMR) analyses, as shown in Figure 1. In the FTIR spectra, all samples exhibited a strong absorption band around 1735 cm^−1^ (highlighted pink region), corresponding to the stretching vibration of ester carbonyl (C=O) groups—clearly indicating the formation of ester linkages via polycondensation. Additional absorption peaks near 2920 and 2850 cm^−1^ (highlighted blue region) were attributed to the asymmetric and symmetric C–H stretching vibrations of methylene (–CH_2_–) groups within the long aliphatic chains, confirming the incorporation of the intended diol and diacid components. The presence of C–O–C stretching vibrations in the region of 1150–1250 cm^−1^ (highlighted green region) further supports the formation of the polyester backbone. A weak, broad band around 3450 cm^−1^ (highlighted yellow region), which is most pronounced in PPCM1010, may be associated with residual hydroxyl groups from unreacted diol end groups, although its intensity decreases in samples with longer chains, suggesting higher molecular weight and conversion efficiency. Similar characteristic peaks have been reported in the literature for aliphatic polyesters [33, 34], confirming that the observed FTIR spectra in the current study indicate successful synthesis of the intended polyester structures.

These FTIR results were confirmed by ^1^H NMR spectra, which showed characteristic signals corresponding to the expected polymer structure (Figure 1b). The resonances at 4.05–4.15 ppm (highlighted green region) were assigned to the methylene protons adjacent to the ester oxygen (–O–C*H*_2_–) [23], while signals at 2.25–2.35 ppm (highlighted red region) are attributed to methylene groups adjacent to the ester carbonyl (–CO–C*H*_2_–) [35]. The signals 1.29 and 1.60 ppm (highlighted blue region) corresponded to the methylene protons of the aliphatic chains [36], with increased intensity observed in PPCM1212 and PPCM1218 due to their extended chain lengths. Hydroxyl end groups are observed at 3.65 ppm (–C*H*_2_OH, highlighted yellow region). The signal for hydroxyl end groups is stronger for PPCM1212 than for PPCM1010 and PPCM1218, which could indicate a lower molecular weight or a higher dispersity; the latter was confirmed by size exclusion chromatography (SEC). Together, the FTIR and ^1^H NMR results conclusively demonstrated the successful synthesis of linear aliphatic polyesters.

The successful formation of PPCMs was further confirmed by SEC analysis. The results are shown in Table 1, and the corresponding differential weight distribution curves are shown in Figure 1c. The number‐average molecular weight (M_*n*_) increased from 12,100 g/mol for PPCM1010 to 15,300 g/mol for PPCM1212, reaching 26,200 g/mol for PPCM1218. A similar trend was observed for the weight‐average molecular weight (M*_w_*), which rose from 22,100 g/mol to 31,600 g/mol and 50 900 g/mol, respectively. This progressive increase in molecular weight can be attributed to the use of longer‐chain diols and diacids in PPCM1212 and PPCM1218, which result in higher repeat unit masses and thus contribute to greater molecular weights, assuming comparable degrees of polymerization across samples. The molecular weight dispersity (*Ð* = M*_w_*/M_*n*_) ranged from 1.84 to 2.07 for the obtained polymers. These values are typical of polyesters synthesized via step‐growth polymerization [37, 38], reflecting a moderate yet acceptable distribution of molecular weights. Although higher molecular weights have been reported in the literature [33, 39], achieving them typically requires precise control of monomer ratios and efficient removal of condensation by‐products under high vacuum and extended reaction times. In this work, the obtained molecular weights are within the typical range for polyesters [40, 41], sufficient for melt‐ or solution‐based processing such as electrospinning [42]. Moreover, blending with PA11 is expected to further improve the mechanical performance of the materials.

**TABLE 1:** Molecular weight characteristics of PPCMs determined by SEC.

Sample	**M** *_**n**_* **, g/mol**	**M** *_**w**_* **, g/mol**	Ð
PPCM1010	12,100	22,100	1.84
PPCM1212	15,300	31,600	2.07
PPCM1218	26,200	50,900	1.95

The phase change properties of the synthesized PPCMs were analyzed by differential scanning calorimetry (DSC), and the results are shown in Table 2, with the corresponding thermograms shown in Figure 1d. All PPCMs exhibited distinct endothermic and exothermic peaks corresponding to melting (*T*_m_) and crystallization (*T*_c_), respectively, confirming their solid–liquid phase change behavior. The melting temperatures increased with the length of the aliphatic chains in the diol and diacid monomers, ranging from 76.2°C for PPCM1010 to 90.6°C for PPCM1218. A similar trend was observed for crystallization temperatures, which rose from 61.6°C in PPCM1010 to 80.9°C in PPCM1218. This behavior is consistent with the enhanced chain regularity and stronger van der Waals interactions in longer‐chain polyester segments, leading to higher thermal stability.

**TABLE 2:** Thermal properties of PPCMs determined by DSC.

Sample	***T***_**c**_ **,°C**	***T***_**m**_ **,°C**	**Δ*H***_**c**_ **, J/g**	**Δ*H***_**m**_ **, J/g)**	ΔT,°C
PPCM1010	61.6	76.2	122	123	14.6
PPCM1212	69.7	81.0	130	136	11.3
PPCM1218	80.9	90.6	148	157	9.7

The phase change enthalpies (Δ*H*_m_ and Δ*H*_c_), which reflect the material's latent heat storage capacity, also increased with chain length. Specifically, the melting enthalpy increased from 123 J/g for PPCM1010 to 157 J/g for PPCM1218, indicating that PPCM1218 can store and release more thermal energy during phase transition. Crystallization enthalpies followed a similar pattern.

Supercooling, defined as the difference between *T*_m_ and *T*_c_ (ΔT), is an important parameter for evaluating the reversibility and efficiency of phase change processes. A lower degree of supercooling is generally desirable for thermal energy storage applications, as it enables more reliable crystallization and faster heat release during cooling cycles [43]. Δ*T* decreased with increasing chain length, from 14.6°C for PPCM1010 to just 9.7°C for PPCM1218. This reduction in supercooling is advantageous, as it allows the PCM to crystallize more rapidly, thereby releasing the stored latent heat more efficiently [44]. The slightly broader melting peak and intermediate supercooling observed for PPCM1212 are consistent with its higher molecular weight dispersity (*Ð* = 2.07), reflecting the presence of both shorter and longer polymer chains within the sample. This effect is typical of step‐growth polyesters and does not compromise the effectiveness of the phase‐change behavior.

PPCM1218 exhibits a phase change enthalpy comparable to several commercially available organic PCMs such as decanoic acid (≈ 160 J/g) [45] and PEG4000 (150–170 J/g) [46]. However, its higher melting temperature (≈ 90.6°C) makes it particularly suitable for medium‐to‐high temperature thermal storage, with potential applications in concentrated solar power systems, waste‐heat recovery, and thermal management of high‐power electronics [47, 48]. Unlike conventional organic PCMs such as paraffins or fatty acids, PPCM1218 is a polyester—rendering it inherently more compatible with textile processing. Thus, it was selected for incorporation into electrospun textile mats for potential energy storage applications.

### 2.2 Phase Change Materials Mats Characterization

#### 2.2.1 Microstructure Study

**FIGURE 2:**
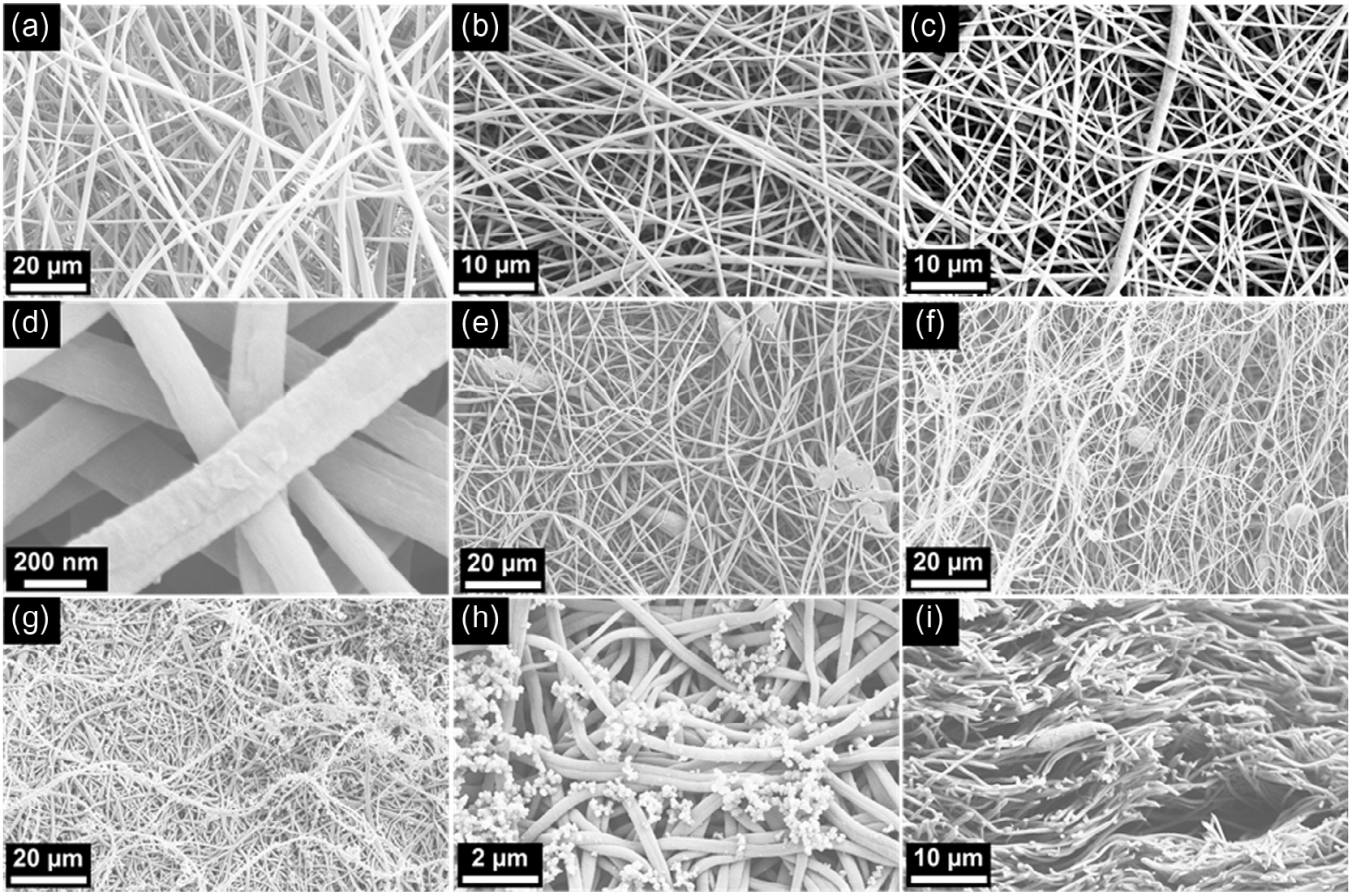
SEM micrographs of the electrospun mats: (a) PA11, (b) ePPCM50, (c,d) ePPCM85, (e) BC@ePPCM85, (f) Gr@ePPCM85, and (g–i) PPy@ePPCM85. All images were captured from the surface of the mats, except image (i), which was taken from the cryo‐fractured cross section. The accelerating voltage was fixed at 5 kV for all samples. Images are representative of at least three randomly selected regions per sample. SEM = Scanning electron microscopy.

Scanning electron microscopy (SEM) was employed to investigate the surface morphology and fiber structure of the electrospun samples. As shown in Figure 2, the pure PA11 electrospun mats revealed well‐defined, continuous nanofibers with uniform morphology. The fibers exhibited smooth surfaces without visible bead defects or fiber breakages, indicating optimal electrospinning conditions for PA11 under the chosen parameters. The diameter distribution appeared consistent, reflecting stable jet formation and uniform solvent evaporation during the spinning process.

The SEM images of the electrospun mats containing PCMs, namely ePPCM50 and ePPCM85, demonstrated that the good spinnability of PA11 was retained despite the incorporation of PPCM1218 (Figure 2b,c). The fibers were continuous and bead‐free, indicating stable fiber formation during electrospinning. No observable phase separation between PA11 and PPCM components was detected, suggesting good compatibility and homogeneous blending of the two constituents within the fibers. However, a slight roughening of the fiber surfaces was observed compared to pure PA11, particularly in the ePPCM85 sample with the higher PCM content. This surface texture change was more pronounced at higher magnifications and is likely attributable to the increased presence of PPCM within the fiber matrix (see Figure 2d). To the best of the authors’ knowledge, there are no prior reports on the electrospinning of PA/polyester blends. Nonetheless, the observed compatibility between PA11 and PPCM1218 is consistent with previous studies on polyester–PA systems developed by melt blending, which have demonstrated strong interfacial adhesion and homogeneous morphology. For example, Li et al. [49] demonstrated that introducing a compatibilizer improved compatibility, mechanical strength, and thermal stability in PLA/PA11 blends due to favorable polyester–PA interactions. Similarly, a hyperbranched polyester compatibilizer enhanced interfacial adhesion and toughness in PA56/PBAT blends via block copolymer formation and hydrogen bonding [50]. Although no compatibilizer was used here, the inherent chemical compatibility between PPCM1218's polyester backbone and PA11 likely enabled uniform dispersion during electrospinning, as supported by the absence of phase separation in SEM images, even at higher PPCM loadings. Due to this inherent compatibility, the sample with the highest PCM content, ePPCM85, was selected for developing photothermal mats, as it exhibits the highest phase change enthalpy, which will be discussed in the following section.

After incorporating photothermal particles, namely BC and Gr, SEM analysis of the BC@ePPCM85 and Gr@ePPCM85 samples revealed that the fibers remained continuous and well‐formed despite the presence of these fillers, as shown in Figure 2e,f. However, some bead‐like defects were observed in both samples, suggesting slight instability during the electrospinning process, likely caused by the addition of particulate fillers. These beads are likely due to localized agglomeration or uneven dispersion of biochar and graphene within the spinning solution, which can influence the solution's viscosity and charge distribution during jet formation. Similar findings were reported for reduced graphene oxide‐containing PA66 electrospun mats, where particle concentrations above 2 wt% led to local agglomerations and bead formation in the fibers [51].

As shown in **Figure S1**, energy‐dispersive X‐ray spectroscopy (EDX) elemental mapping of ePPCM85 fibers revealed the distribution of carbon (C) and oxygen (O), which originated from both PPCM and PA11. Nitrogen (N) from the PA11 amide groups was not clearly observed in the unmodified mats, likely due to coverage by the polyester matrix. After PPy polymerization, nitrogen was detected in the maps of PPy@ePPCM85, along with morphological features such as spherical particles on the fiber surfaces (Figure 2g,h), confirming successful surface modification and deposition of PPy [52]. The surface appeared smooth and uniform, with minimal particulate presence, suggesting controlled pyrrole polymerization and deposition that preserved structural integrity and maintained clear transport pathways [53]. The SEM analysis of the fiber cross sections revealed the presence of similar spherical particles embedded within the fiber interior (Figure 2i), demonstrating that PPy was not only deposited on the surface but also penetrated and polymerized inside the fibers. This dual localization suggests that the pyrrole monomer effectively diffused into the fiber matrix prior to polymerization, leading to a more integrated composite structure.

#### 2.2.2 Mechanical Properties

**FIGURE 3:**
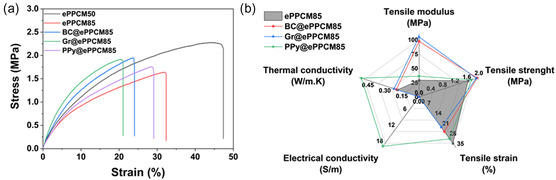
Representative stress–strain curves of the mats. (a) Comparison of the mechanical properties, thermal conductivity, and electrical conductivity of ePPCM85 and the corresponding photothermal‐enhanced mats. Data in (b) are derived from three to five independent experiments (*n* = 3–5) and are reported as mean ± SD. Statistical significance in (b) was evaluated using one‐way ANOVA followed by Tukey's post hoc test ( *p* < 0.05). ANOVA = Analysis of variance.

The mechanical properties of the electrospun mats were investigated to evaluate their suitability for practical applications. Representative stress–strain curves are shown in Figure 3a, while the corresponding mechanical properties, including tensile modulus, tensile strength, and tensile strain (elongation at break), are shown in Table 3. Comparing the two base formulations, ePPCM50 and ePPCM85, an increase in PPCM concentration from 50 wt% to 85 wt% led to a decrease in tensile modulus (from 37 ± 2 MPa to 29 ± 3 MPa) and tensile strength (from 2.27 ± 0.31 MPa to 1.64 ± 0.25 MPa), along with a reduction in tensile strain (from 47 ± 2% to 32 ± 3%). This decline can be attributed to the lower intrinsic mechanical strength of PPCM compared to PA11, a polymer well known for its excellent mechanical properties. Reinforcing effects of PAs on brittle polymers have also been reported by Razavizadeh et al. [54], where blending PA6 with polystyrene significantly improved the tensile strength of the electrospun webs. Similarly, Mousavi et al. [55] observed a significant improvement in the mechanical properties of chitosan fibers after the incorporation of PA6.

**TABLE 3:** Mechanical properties, electrical conductivity, and thermal conductivity of electrospun composite mats.

Sample	Tensile modulus, MPa	Tensile strength, MPa	Tensile strain,%	Electrical conductivity, µS/m	Thermal conductivity, W/m·K
ePPCM50	37 ± 2	2.27 ± 0.31	47 ± 2	1.5 ± 0.2	0.19 ± 0.01
ePPCM85	29 ± 3	1.64 ± 0.25	32 ± 3	1.7 ± 0.3	0.20 ± 0.01
BC@ePPCM85	97 ± 5	1.94 ± 0.33	24 ± 2	1.8 ± 0.2	0.22 ± 0.02
Gr@ePPCM85	105 ± 4	1.91 ± 0.24	21 ± 2	4.5 ± 0.3	0.27 ± 0.02
PPy@ePPCM85	36 ± 2	1.75 ± 0.25	29 ± 3	(18.5 ± 1.1) × 10^6^	0.51 ± 0.03

*Note:* Data are presented as mean ± SD from three to five independent experiments (*n* = 3–5). Statistical significance was determined using one‐way ANOVA followed by Tukey's post hoc test ( *p* < 0.05).

The introduction of 10 wt% biochar (BC@ePPCM85) or graphene (Gr@ePPCM85) into the ePPCM85 matrix resulted in a notable increase in tensile modulus compared to the unmodified ePPCM85 sample. Specifically, the modulus increased from 29 ± 3 MPa for ePPCM85 to 97 ± 5 MPa and 105 ± 4 MPa for the BC and graphene composites, respectively. Similarly, the tensile strength increased from 1.64 ± 0.25 MPa to 1.94 ± 0.33 MPa and 1.91 ± 0.24 MPa for the same samples. This modest improvement, despite the substantial rise in modulus, is likely due to partial filler aggregation and reduced polymer chain extensibility, which slightly limits load transfer under tensile stress. Such behavior, common in electrospun mats reinforced with rigid nanoparticles, also accounts for the observed decrease in elongation at break. For example, in electrospun polystyrene mats reinforced with cellulose nanocrystals, an increase in nanoparticle content led to higher tensile modulus accompanied by a reduction in elongation at break [56], supporting the stiffening–ductility trade‐off observed here.

This enhancement reflects the inherent stiffness of the biochar and graphene particles, which act as reinforcing fillers within the polymer matrix. Their incorporation likely improved load transfer and restricted polymer chain mobility, resulting in a stiffer material. Additionally, these nanofillers likely act as stress‐bearing components and contribute to improved mechanical reinforcement through strong interfacial interactions and physical entanglement with the polymer chains. Similar enhancements in mechanical properties have been observed in PA‐based mats reinforced with graphene nanoparticle [57, 58, 59]. It is worth mentioning that SEM images (Figure 2e,f) revealed the presence of bead‐like structures in the BC‐ and Gr‐containing mats, which may result from local aggregation or surface tension effects during electrospinning. While these beads could slightly disrupt fiber uniformity, they did not significantly compromise the mechanical performance, likely due to the dominant reinforcing role of the fillers and the overall integrity of the fiber network.

The PPy@ePPCM85 sample exhibited a tensile strength of 1.75 ± 0.25 MPa, which is slightly higher than that of the uncoated ePPCM85 mat (1.64 ± 0.25 MPa), while the tensile strain decreased slightly from 32 ± 3% to 29 ± 3% following PPy surface polymerization. However, the tensile modulus increased significantly from 29 ± 3 MPa to 36 ± 2 MPa, corresponding to a 25% enhancement in stiffness. Oliveira et al. [60] also reported similar trends, where the incorporation of PPy into electrospun mats led to an increase in tensile modulus of polylactic‐co‐glycolic acid. Similarly, in our previous work [16], we observed that PPy coating slightly enhanced the mechanical properties of the PA11/decanoin acid mats, mainly through additional hydrogen bonding. However, Pérez‐Nava et al. [59] demonstrated that PPy deposition on electrospun poly(vinyl alcohol) nanofibers substantially enhanced the mechanical performance, with improvements observed in Young's modulus, tensile strength, and elongation at break. Nevertheless, the mechanical properties of PPy@ePPCM85 fall well within the typical range reported for electrospun mats used in energy storage applications such as flexible supercapacitors and batteries, indicating that the material maintains sufficient mechanical integrity for practical use [61, 62, 63]. In addition, **Video S1** further demonstrates the flexibility of the PPy@ePPCM85 mats under bending and handling.

#### 2.2.3 Electrical and Thermal Conductivity

The electrical and thermal conductivities of the electrospun composite mats were measured to evaluate their functional performance, and the results are shown in Table 3. The base mats, ePPCM50 and ePPCM85, exhibited very low electrical conductivities on the order of µS/m (1.5 ± 0.2 µS/m and 1.7 ± 0.3 µS/m, respectively), consistent with the intrinsic insulating nature of the polyester and PA11 components and in line with values reported in the literature [64, 65]. Their thermal conductivities were similarly low, at 0.19 ± 0.01 W/m·K and 0.20 ± 0.01 W/m·K, reflecting the porous and polymeric structure of the mats. These values are in good agreement with those reported in the literature for PA [66] and polyester [67] fibers.

The incorporation of 10 wt% biochar (BC@ePPCM85) into the ePPCM85 matrix led to a modest increase in thermal conductivity but no significant changes in electrical conductivity. Specifically, the thermal conductivity increased to 0.22 ± 0.02 W/m·K, while the electrical conductivity remained low at 1.8 ± 0.2 µS/m. More pronounced enhancements were observed for the Gr@ePPCM85 composite, where the thermal conductivity reached 0.27 ± 0.02 W/m·K and the electrical conductivity increased to 4.5 ± 0.3 µS/m. Although the incorporation of graphene increased the electrical conductivity by more than two orders of magnitude compared to the base mats, the value (4.5 ± 0.3 µS/m) still lies at the lower end of the semiconductor regime and is characteristic of an insulating polymer matrix with only limited conductive pathways. Several reports on graphene‐based polymer composites have demonstrated significantly higher electrical conductivities even at relatively low graphene loadings [68], where the fillers reach the percolation threshold. In contrast, the relatively low conductivity measured in this study may be attributed to the agglomeration of graphene particles, as suggested by the formation of bead‐like structures observed in the SEM images (see Figure 2f). Reducing the graphene loading could help to minimize particle agglomeration; however, under such conditions, the color of the mats would not be sufficiently dark (see **Figure S2**) to be suitable for photothermal applications.

Notably, the PPy@ePPCM85 mat exhibited a dramatic increase in electrical conductivity, reaching 18.5 ± 1.1 S/m, several orders of magnitude higher than all other samples. This substantial enhancement is attributed to the *in situ* polymerization of PPy both on the surface and within the fiber interiors. The continuous, bead‐free, and homogeneous fiber morphology of the substrate mats (ePPCM85), confirmed by SEM (Figure 2), provided an ideal scaffold for uniform PPy deposition, facilitating the formation of a continuous conductive network. This well‐defined PPy network significantly enhances electron transport, effectively transforming the originally insulating mat into a highly conductive composite. Moreover, the thermal conductivity of the PPy‐coated mats doubled compared to the other samples, reaching 0.51 ± 0.02 W/m·K, likely due to improved phonon transport through the rigid and conjugated PPy domains. This enhancement is attributed to the formation of a continuous PPy network across the fibers, which facilitates phonon transport and establishes efficient thermal pathways throughout the mat.

Comparable improvements have been reported for PPy polymerization on flax fiber [45], wood sponge [69], Janus cotton fabric [70], and polyurethane foam [71], particularly in the context of photothermal applications. It should be emphasized that a wide range of thermal (≈ 0.3–12 W/m·K) and electrical (≈ 10^2^–10^3^ S/m) conductivity values have been reported in the literature for conductive PCM composites designed for photothermal and electrothermal conversion applications [72, 73]. The values obtained for the PPy@ePPCM85 sample in the present work fall well within this reported range, underscoring its potential for efficient thermal energy harvesting, storage, and thermal management applications.

#### 2.2.4 Phase Change Properties

**FIGURE 4:**
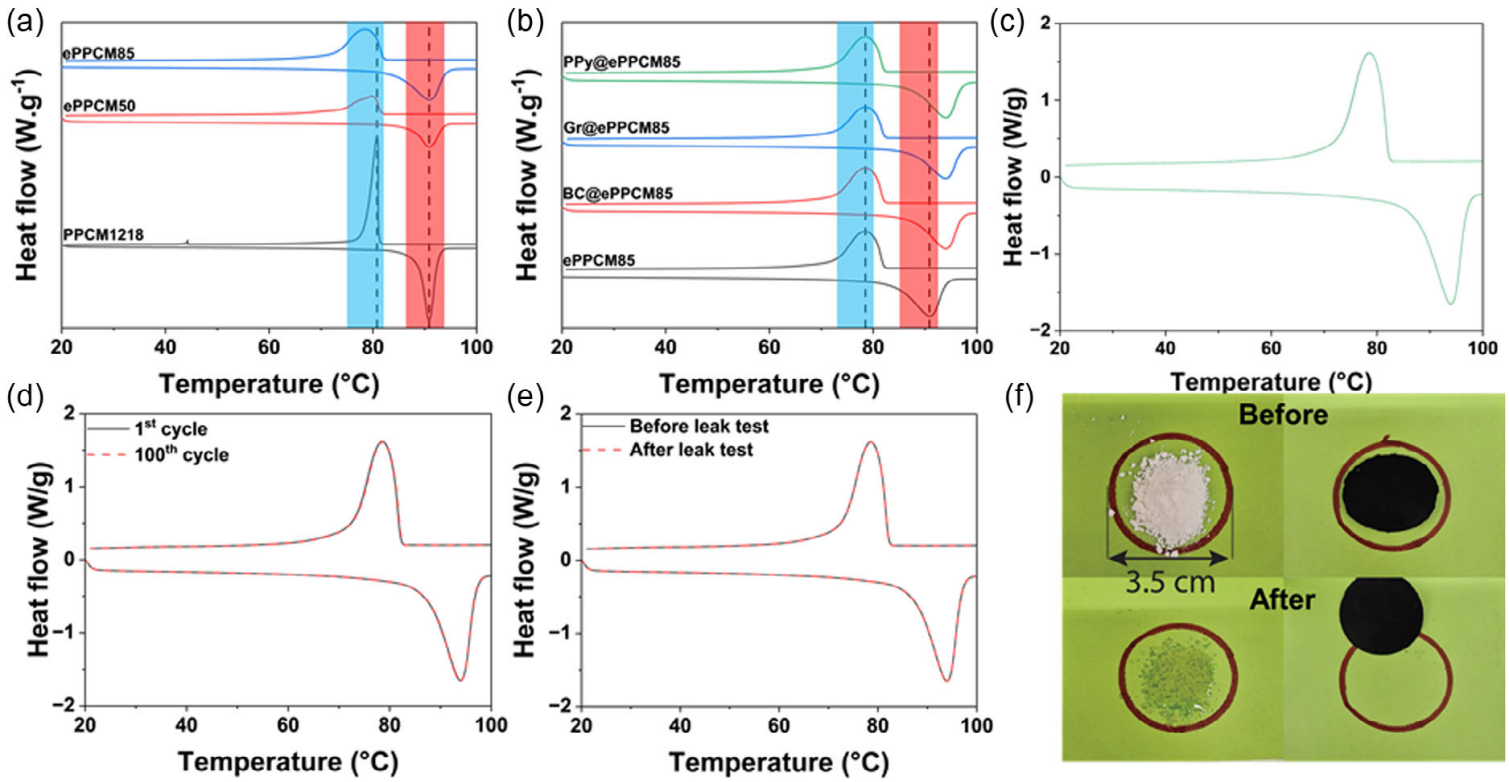
Phase change properties of the samples analyzed by DSC. (a) DSC thermograms of plain PPCM1218 and the corresponding electrospun mats with 50% and 85% PCM content. (b) DSC curves of ePPCM85 and the photothermal‐enhanced mats. (c) Thermal reliability of PPy@ePPCM85 over 100 DSC cycles. (d) Comparison of the 1^st^ and 100^th^ DSC cycles of PPy@ePPCM85. (e) DSC thermograms of PPy@ePPCM85 before and after the leakage test. (f) Digital photographs of PPCM85 and PPy@ePPCM85 before and after the leakage test. All measurements were conducted on representative samples, and the data shown are representative of the obtained results. DSC = Differential scanning calorimetry; PCM = phase change material.

The phase change behavior of the electrospun mats was investigated using DSC, and the results are shown in Figure 4a and Table 4. ePPCM50 exhibited Δ*H*_c_ and Δ*H*_m_ values of 74 and 77 J/g, respectively—approximately half of the original PPCM1218 values, consistent with the reduced PCM content. Increasing the PPCM content to 85 wt% in ePPCM85 proportionally enhanced the energy storage capacity, yielding Δ*H*_c_ of 126 J/g and Δ*H*_m_ of 132 J/g. On the other hand, the phase change temperatures did not change significantly after encapsulating the PPCM in the PA11 matrix, with only slight reductions observed (e.g., *T*_m_ shifted from 90.6°C in PPCM1218 to 90.1°C in ePPCM85). These results indicate good compatibility and uniform mixing between PPCM and PA11, suggesting that the supporting polymer did not disrupt the crystallization or melting behavior of the PCM [74]. The minor shifts in temperatures confirm that PA11 served as an effective electrospinning aid without significantly restricting the phase change properties, thereby preserving the thermal energy storage functionality of the system [75, 76].

**TABLE 4:** Phase change properties of fabricated mats.

Sample	***T***_**c**_ **,°C**	***T***_**m**_ **,°C**	**Δ*H***_**c**_ **, J/g**	**Δ*H***_**m**_ **, J/g**	ΔT,°C
ePPCM50	79.8	90.8	74	77	11.0
ePPCM85	78.3	90.1	126	132	11.8
BC@ePPCM85	78.1	94.1	115	122	16
Gr@ePPCM85	78.2	94.0	114	121	15.8
PPy@ePPCM85	78.2	94.3	110	116	16.1
Leakage test results of PPy@ePPCM85 before and after 2 h heat treatment in an oven at 120°C					
Before heating	78.1	94.1	110	116	16.0
After heating	78.1	94.1	110	116	16.0
Durability results of PPy@ePPCM85 based on 100 DSC thermal cycles: comparison of phase change properties between the 1^st^ and 100^th^ cycles.					
1^st^ cycle	78.2	94.3	110	116	16.1
100^th^ cycle	78.2	94.3	110	116	16.1

Further modifications to ePPCM85 with photothermal additives—namely biochar, graphene, and PPy coatings—had notable effects on the phase change behavior, as shown in Figure 4b and Table 4. All modified mats (BC@ePPCM85, Gr@ePPCM85, and PPy@ePPCM85) exhibited a slight further decrease in enthalpy values. For example, Δ*H*_m_ decreased from 132 J/g in ePPCM85 to 116 J/g in PPy@ePPCM85. This reduction is consistent with the dilution of the PPCM component in the overall formulation, as detailed in the experimental section, where the added photothermal particles occupy part of the matrix volume without contributing to latent heat storage. Comparable or higher enthalpies have been reported for PCM composites designed for photothermal applications such as paraffin wax/thermoplastic elastomer/carbon nanotube composites (180 J/g) [77], stearic acid/multiwalled carbon nanotubes/hexagonal boron nitride composites (147 J/g) [78], and multifunctional Ni‐MOF/MXene/paraffin composites (177 J/g) [79]. Nonetheless, the Δ*H*_m_ of PPy@ePPCM85 remains within the range reported for flexible PCM composites for wearable thermal management [80, 81].

Interestingly, upon the incorporation of these additives, *T*_c_ remained relatively unchanged, indicating that nucleation during cooling was not significantly hindered. However, *T*_m_ showed a noticeable upward shift. For instance, *T*_m_ increased from 90.1°C in ePPCM85 to 94.3°C in PPy@ePPCM85. This shift could be attributed to increased interfacial interactions between the PCM domains and the added particles or coatings, which may have altered the melting kinetics and crystal growth behavior, while restricting PCM chain mobility in the molten state, thereby stabilizing the liquid phase and delaying the onset of melting. Similar upward shifts in *T*_m_ have been reported for microencapsulated stearic acid with SiO_2_ shells, where interfacial interactions delayed the melting transition [82], and for lauric acid/SiO_2_ composites, where shell/core interactions altered phase transition behavior [83]. More generally, confinement of PCMs in porous supports has been shown to shift phase‐change temperatures due to substrate–PCM interactions and restricted crystal growth [84, 85, 86]. In contrast, a reduction in the melting point of PEG PCMs has been reported after the incorporation of graphene particles. This effect was attributed to the higher thermal conductivity of graphene, which accelerated the melting process, while strong PEG–graphene interactions slowed down crystallization during cooling [87].

Unlike many reports where the incorporation of fillers or nanoparticles into PCMs effectively suppresses supercooling [88, 89, 90], it increased significantly to 15.8–16.1°C upon modification with biochar, graphene, or PPy coatings. This increase originated primarily from the upward shift in *T*_m_, while *T*_c_ remained essentially unchanged (≈ 78°C). As discussed earlier, the observed behavior can be attributed to stronger interfacial interactions between the PCM domains and the added particles or coatings, which stabilize the molten state and delay nucleation.

#### 2.2.5 Thermal Durability and Stability

To assess long‐term thermal reliability, PPy@ePPCM85 was subjected to 100 continuous DSC heating–cooling cycles. As shown in Figure 4c,d and shown in Table 4, the phase change parameters after the 100^th^ cycle were essentially unchanged from the first cycle, with no measurable shifts in transition temperatures or enthalpy values. This preservation of enthalpy and crystalline structure integrity confirms the material's ability to endure repeated melting and crystallization without degradation [91, 92].

Thermal stability and shape integrity during phase transition were further examined through a leakage test, where PPy@ePPCM85 was heated at 120°C for 2 h. DSC analysis before and after treatment (Figure 4e) revealed no significant changes in transition temperatures or latent heats, validating both structural stability and PCM encapsulation. Digital photographs of PPy@ePPCM85 and neat PPCM85 before and after testing (Figure 4f) further highlight this contrast, showing that the electrospun mat exhibited no leakage or deformation, whereas the pure PPCM85 completely melted. These observations indicate that PA11 effectively stabilized the PPCM within the fibrous matrix, thereby preventing leakage and flow during the molten state. Taken together, these results demonstrate that the electrospun and surface‐modified architecture of PPy@ePPCM85 ensures robust thermal confinement and long‐term durability, making it a promising candidate for sustainable thermal energy storage applications.

#### 2.2.6 Photothermal Performance

**FIGURE 5:**
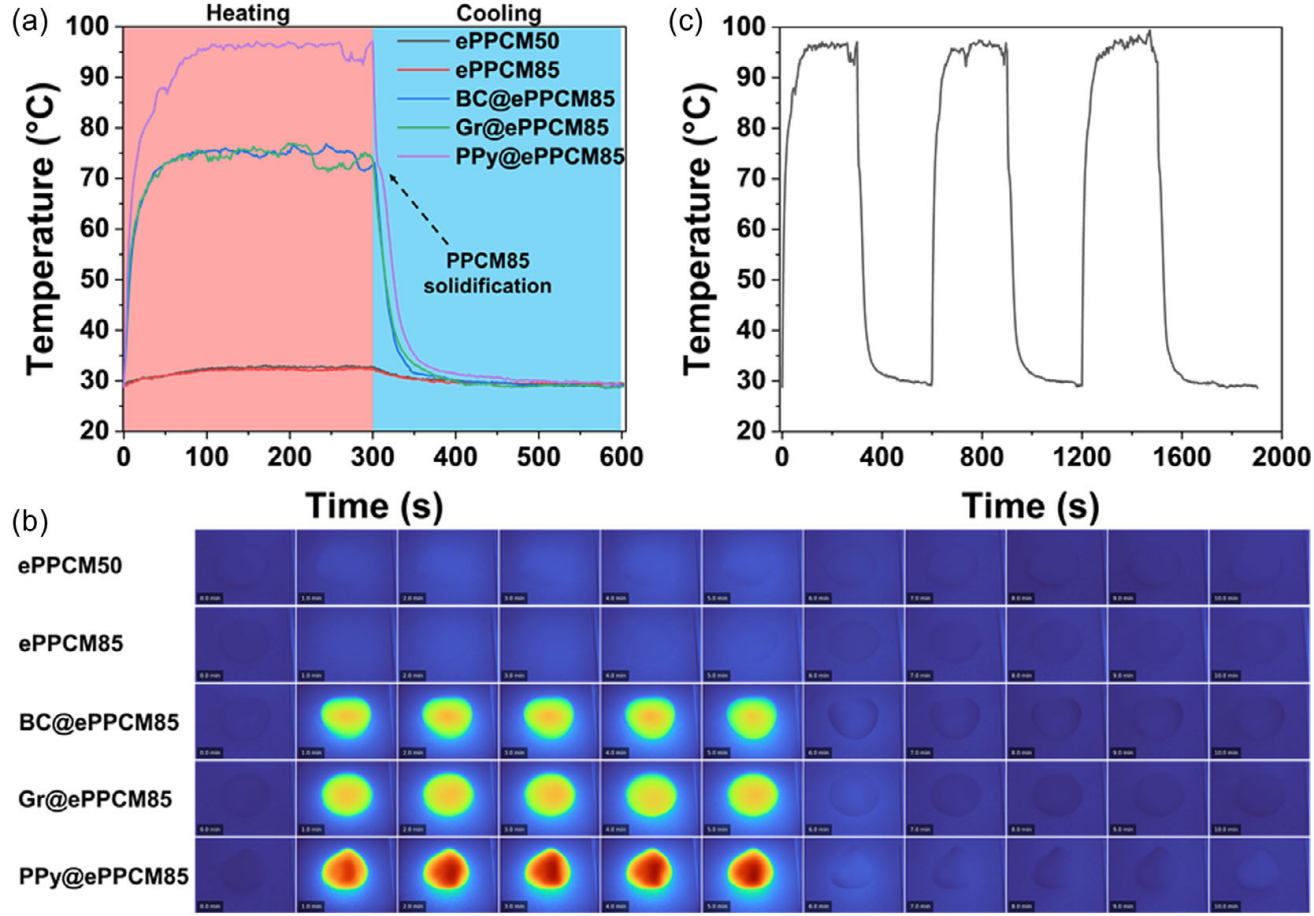
Photothermal performance of the samples under simulated solar irradiation (1.5 sun) with 300 s heating followed by 300 s cooling. (a) Maximum surface temperature profiles comparing all samples. (b) Infrared thermal images capturing the dynamic heating and cooling phases of all samples. (c) Stability and repeatability demonstrated by the maximum surface temperature of PPy@ePPCM85 over three consecutive heating–cooling cycles. All measurements were conducted on representative samples, and the data shown are representative of the obtained results.

The development of flexible textiles with photothermal properties has attracted considerable attention for applications in wearable electronics, smart thermal management systems, and solar energy harvesting [17, 93]. Integrating photothermal functionality into lightweight, breathable, and deformable textile platforms enables efficient conversion of solar energy into heat, which is particularly beneficial for passive heating, personal thermal regulation, and thermal energy storage [94]. It can also find uses in water‐free sunlight‐driven cleaning and disinfection of fabrics. To assess the photothermal performance of the developed mats, samples were exposed to 1.5 sun radiation for 300 s, followed by a 300‐second cooling period after switching off the light source. The resulting temperature profiles are shown in Figure 5a, and the corresponding thermal camera images are shown in Figure 5b and **Video S2**.

In the base electrospun mats (ePPCM‐50 and ePPCM‐85), only a modest temperature increase was observed, with the maximum surface temperatures remaining below 40°C. This behavior is consistent with their inherently low optical absorption and limited thermal conductivity [95, 96]. Since these samples contain no additional photothermal agents, the absorbed solar energy is minimal, and the resulting heat is not efficiently conducted. As expected, the PPCM1218 encapsulated in these mats remained solid, as the temperature did not approach its melting point (ca. 90°C).

In contrast, the incorporation of conductive photothermal fillers, BC and Gr, significantly enhanced the solar‐to‐thermal energy conversion. For both BC@ePPCM85 and Gr@ePPCM85, the surface temperatures increased more rapidly and reached maximum values between 55 and 65°C within the first few minutes of irradiation. This improvement can be attributed to the well‐documented photothermal performance of both biochar [46, 97] and graphene [98]. Biochar, with its high carbon content and porous structure, exhibits strong light absorption across a broad spectrum and efficient nonradiative decay pathways, making it an effective photothermal agent [99, 100]. Similarly, graphene is renowned for its exceptional optical absorption, high thermal conductivity, and rapid electron–phonon interactions, which enable efficient photothermal conversion. These intrinsic properties of biochar and graphene facilitate more effective heat generation and distribution upon solar irradiation, thereby improving the overall photothermal response of the electrospun mats. However, despite this improvement, the rise in temperature was still insufficient to melt the PCM fully. This suggests that while the fillers improve thermal response, the photothermal effect remains moderate and does not reach the threshold for triggering the phase change of PPCM1218 under the applied sunlight conditions.

Notably, the PPy‐coated mat (PPy@ePPCM85) demonstrated the most effective and robust photothermal response. The surface temperature quickly exceeded 90°C, surpassing the melting point of the PPCM and indicating successful activation of the thermal energy storage process. Infrared images (Figure 5b) confirmed that the PCM underwent a phase transition, evidenced by localized heat accumulation and pronounced color changes in the thermal images. Furthermore, no heat spreading beyond the mat region was detected, confirming that the fibrous, PA11‐reinforced structure effectively confined the molten PCM. These results demonstrate that PPy@ePPCM85 can reliably store and release thermal energy without leakage during repeated heating cycles. During the subsequent cooling step, a shoulder around 75°C was observed (see black‐dashed arrow), which can be attributed to the solidification of the PCM and the associated release of latent heat. This behavior highlights the reversible thermal energy storage capability of the material.

This enhanced performance is attributed not only to the strong solar absorption capacity and the high photothermal conversion efficiency (*η*) of 92.3% provided by the PPy coating but also to the significantly higher thermal conductivity of this sample (0.51 W/m·K), which is more than double that of the uncoated counterparts. The improved thermal conductivity facilitates rapid and uniform heat transfer throughout the fibrous network, enhancing the efficiency of heat storage and release, and ensuring a more responsive and stable photothermal behavior. Such performance trends are in line with earlier studies demonstrating PPy's effectiveness in enhancing photothermal conversion and thermal transport in PCMs, as shown in Table 5.

**TABLE 5:** Summary of reported studies on PPy‐modified PCMs, highlighting photothermal conversion efficiency.

Matrix	Photothermal filler	Photothermal conversion efficiency,%	Reference
Wood and paraffin wax	PPy and layered double hydroxide	96.4	[101]
Multishell melamine–formaldehyde resin@octadecane microcapsule	PPy and silver nanoparticles	94.3	[102]
Epoxy resin, delignified wood, and PEG	PPy	91.9	[103]
Melamine formaldehyde and PEG	PPy‐modified halloysite	90.36	[104]
Yeast, capric acid, and lauric acid	PPy	53.3	[105]
PPy aerogel and paraffin wax	PPy	53.3	[106]

To evaluate the photothermal durability and cycling stability of the PPy@ePPCM‐85 mat, the solar irradiation experiment was repeated for three consecutive cycles. As shown in Figure 5c, the thermal profiles remained consistent over all cycles, with negligible performance degradation, demonstrating the excellent stability of the PPy coating and the structural integrity of the composite mat under repeated solar exposure. This confirms that the PPy modification not only enhances the initial photothermal response but also contributes to the long‐term durability of the textile in potential smart and energy‐harvesting textile applications. Taking into account that these fiber mats can rapidly heat to temperatures of more than 70°C, they can find new uses of sunlight in photothermal inactivation of many bacteria and viruses, including SARS‐CoV‐2, for instance, in protective face masks [107].

## 3 Conclusion

This work presents a systematic strategy for designing flexible, multifunctional electrospun mats that integrate polyester‐based PCMs with photothermal and structural reinforcement. By tailoring monomer chain lengths, PPCM1218 was identified as the optimal candidate due to its high latent heat (157 J/g), elevated melting temperature (90.6°C), and thermal reliability. Incorporation into electrospun fibers with PA11 not only enabled stable, bead‐free morphologies but also effectively suppressed PCM leakage, demonstrating strong compatibility between the polyester backbone and the PA matrix. This synergy allowed the mats to achieve high enthalpies (up to 132 J/g in ePPCM85) while maintaining sufficient mechanical flexibility for textile applications. Further enhancement with photothermal modifiers revealed the crucial role of conductive polymer coatings compared to carbon fillers. While biochar and graphene increased stiffness and slightly improved thermal conductivity, their photothermal response was insufficient to activate phase change under simulated sunlight. In contrast, PPy@ePPCM85 achieved a step change in performance, where thermal conductivity doubled to 0.51 W/m·K, electrical conductivity increased by several orders of magnitude (18.5 S/m), and solar irradiation drove surface temperatures above 90°C, triggering full PCM melting. Infrared thermal imaging confirmed localized heating and confined phase transitions without leakage, underscoring the structural integrity provided by PA11. Durability assessments further highlighted the robustness of the system. PPy@ePPCM85 preserved its transition temperatures and enthalpy values over 100 DSC cycles, with no observable degradation. Leakage tests confirmed complete retention of PCM, in stark contrast to neat PPCM, which melted and deformed. Moreover, repeated photothermal cycles yielded consistent heating–cooling profiles, confirming stable and reversible latent heat storage. Altogether, these findings demonstrate that electrospun PA11/PPCM mats with PPy coatings provide a scalable, sustainable, and durable platform for solar‐activated energy storage textiles.

## 4 Experimental

### 4.1 Materials

1,10‐decanediol (C10, purity > 97.0%, GC), 1,12‐dodecanediol (C12, purity > 99.0%, GC), sebacic acid (C10, purity > 98.0%, GC), dodecanedioic acid (C12, purity > 99.0%, GC), graphene nanoplatelets (6–8 nm thickness, 25 µm width), 1,1,1,3,3,3‐hexafluoropropan‐2‐ol (HFIP, purity > 99.0% (GC)), and pyrrole (purity > 99.0%, GC) were procured from Tokyo Chemical Industry (TCI). 1,18‐octadecanedioic acid (C18, purity > 98.0%) was supplied by Cathay Biotech Company, China. Anhydrous toluene (purity 99.8%), reagent‐grade iron(III) chloride (purity 97%), tin(II) 2‐ethylhexanoate (92.5–100.0%), chloroform (for analysis EMSURE ACS, ISO, Reag. Ph Eur), and deuterated chloroform (CDCl_3_, 99.8 atom % D) were obtained from Sigma‐Aldrich. PA11, FMNO grade (*M*_n_ ≈  20,000 g/mol, M*_w_* ≈  40,000 g/mol), was provided by Arkema. Biochar was prepared from wood chips following the method described in our previous study [108].

### 4.2 Polyester Phase Change Material Synthesis

PPCMs were synthesized through a polycondensation reaction involving different diols and diacids with varying chain lengths (diol:diacid, C10:C10, C12:C12, and C12:C18). To ensure optimal reaction conditions, a slight excess of diol was used. The diol and diacid were added to a three‐neck round‐bottom flask under a nitrogen atmosphere to prevent oxidation. Tin(II) 2‐ethylhexanoate (Sn(Oct)_2_, 0.05 wt% relative to total monomer mass) was introduced as a catalyst to accelerate the reaction. The reaction temperature was initially raised to 150°C and maintained for 8 h under a continuous nitrogen flow to facilitate esterification, followed by an increase to 220°C for an additional 8 h to achieve high‐molecular‐weight polyesters through condensation. After completion, the reaction mixture was cooled to 80°C, and toluene was added to dissolve the synthesized PPCM. The dissolved product was then precipitated in ethanol and washed several times with ethanol to remove impurities such as unreacted monomers and oligomers. The purified PPCM was dried under vacuum at 60°C. The synthesized PPCMs were designated as PPCM1010, PPCM1212, and PPCM1218 based on the chain lengths of the diols and diacids used.

### 4.3 Electrospinning of Mats

Electrospun mats incorporating PPCM were fabricated using a custom‐built electrospinning setup. Among the synthesized PPCMs, PPCM1218 was selected for electrospinning because of its comparatively higher phase change enthalpy. To enhance spinnability, PA11 was employed. A specified amount of PA11 was dissolved in HFIP, while PPCM1218 was dissolved separately in the same solvent. The two solutions were then combined in weight ratios of 50:50 and 85:15 (PPCM1218:PA11), yielding formulations labeled as ePPCM50 and ePPCM85, respectively. The electrospinning solution was optimized at 15 wt% and processed using a stainless‐steel needle (gauge 20) at 15–20 kV, with a flow rate of 10–20 μL/min and a 15 cm needle‐to‐collector distance. The resulting nanofibrous mats were collected on a rotating drum (200 rpm) and analyzed for morphology and thermal performance.

The mat with the higher PPCM content, e.g., ePPCM85, was selected for further enhancement of its photothermal properties. Three different modification strategies were employed. In the first approach, the mat was mixed with 10 wt% biochar to improve photothermal absorption and was coded as BC@ePPCM85. In the second approach, the mat was integrated with 10 wt% graphene to enhance thermal conductivity and photothermal response, coded as Gr@ePPCM85. In the third approach, surface polymerization was carried out by impregnating the mat with pyrrole monomer. In the first two approaches, BC or graphene was dispersed in HFIP using a probe sonicator for 30 min, with the vial surrounded by ice to minimize solvent evaporation. Subsequently, the synthesized PPCM was dissolved in the graphene dispersion and added to the prepared PA11 solution. The mixture was thoroughly blended to achieve uniformity before being used for electrospinning. In the third approach, the mat was impregnated with pyrrole monomer (10% of the mat's mass) and kept at 5°C for 4 h to ensure uniform absorption of the monomer. A solution of iron(III) chloride in 1 M HCl, maintained at 5°C, was prepared as the oxidizing agent. The impregnated mat was immersed in this solution for 4 h to facilitate polymerization. Afterward, the mat was removed and thoroughly washed with water and ethanol to eliminate unreacted monomers and residual iron chloride, resulting in the formation of PPy@ePPCM85. Digital photographs of all the mats are shown in **Figure S3**.

### 4.4 Characterizations

#### 4.4.1 Fourier‐Transform Infrared and Proton Nuclear Magnetic Resonance Characterization

FTIR spectra were recorded using a PerkinElmer FTIR spectrometer equipped with a diamond ATR accessory to identify the characteristic functional groups present in the PPCMs. The scans were collected in the wavenumber range of 4000–500 cm^−1^ with a resolution of 4 cm^−1^.

The chemical structure of the synthesized PPCMs was further verified using ^1^H NMR spectroscopy. Spectra were recorded on a Bruker AV NEO 400 MHz NMR spectrometer at room temperature. Samples were dissolved in deuterated chloroform (CDCl_3_) at a concentration of ≈ 10 mg/mL. Tetramethylsilane (TMS) was used as an internal chemical shift reference (*δ* = 0 ppm). Each spectrum was acquired with 16–32 scans and a relaxation delay of 2 s to ensure optimal signal‐to‐noise ratio and accurate integration. The resulting chemical shifts (δ) were reported in parts per million (ppm), and peak assignments were made based on the expected chemical environments of protons in aliphatic polyester structures.

#### 4.4.2 Molecular Weight Characterization

The number‐average molecular weight (*M*_n_), weight‐average molecular weight (*M*_w_), and dispersity (*Ð* = *M*_w_/*M*_n_) of the synthesized PPCMs were determined by SEC using an Agilent system. The system was equipped with an Agilent 1100 HPLC G1311A quaternary pump, an Agilent 1100 ALS G1313A autosampler, two PLgel Mixed‐C columns (Agilent Technologies, 5 µm, 300 × 7.5 mm) connected in series with a matching guard column, and an Agilent 1200 G1362A refractive index detector. Chloroform (HPLC grade) was used as the mobile phase at a flow rate of 1.0 mL/min. The SEC system was calibrated using polymethyl methacrylate standards. The PPCM samples were dissolved in chloroform at a concentration of 2 mg/mL, filtered through 0.45 µm PTFE syringe filters, and injected into the system without further treatment.

#### 4.4.3 Microstructure

SEM was utilized for the morphological analysis of the electrospun mats. A Zeiss Sigma VP SEM was used to assess fiber uniformity and surface modifications. The samples were coated with a thin layer of gold or platinum to improve conductivity and enhance imaging resolution. SEM images were captured at various magnifications to observe the surface morphology, fiber diameter, and any changes in structure due to particle incorporation or surface polymerization. The SEM was operated at an accelerating voltage of 5 kV. EDX was performed on the ePPCM85 mat before and after PPy surface polymerization to verify the presence and distribution of PPy on the fiber surfaces.

#### 4.4.4 Mechanical Properties

The tensile properties of the electrospun mats were measured using a universal testing machine (Instron 4204, Instron Corp., USA) equipped with a 100 N load cell. The mats were cut into rectangular strips with dimensions of 40 mm × 10 mm, and the average thickness of each sample was determined using a digital micrometer. Prior to testing, all specimens were conditioned at 25 ± 2°C and 50 ± 5% relative humidity for at least 24 h. The tensile tests were performed at a constant crosshead speed of 5 mm/min, and the gauge length was set to 20 mm. The stress–strain curves were recorded and analyzed using Bluehill software. Each test was repeated at least five times, and the average values of tensile strength, elongation at break, and Young's modulus were reported.

#### 4.4.5 Electrical Conductivity Measurement

The electrical conductivity of the modified mats was measured using a four‐point probe system from Ossila, UK, at room temperature. The measurements were recorded as the average of 10 readings taken at room temperature.

#### 4.4.6 Thermal Conductivity Measurement

Thermal conductivity was measured using a Hot‐Disk Thermal Constants Analyzer (model) to evaluate heat transfer efficiency in the mats. The measurements were performed in accordance with the transient plane source (TPS) method, which applies a heated sensor between two layers of the sample to determine the thermal conductivity. The tests were conducted at room temperature, and data were collected in triplicate to ensure accuracy.

#### 4.4.7 Leakage Test

The leakage resistance of PPy@ePPCM85 was evaluated by thermal treatment above the PCM's melting point. The sample was placed on filter paper and heated in an oven at 120°C for 2 h. Photographs were taken before and after the heat treatment to visually assess any leakage of the encapsulated PCM. The presence of stains or discoloration on the filter paper was used as an indicator of leakage. A pure PPCM sample was used as a control to benchmark the leakage behavior against the composite mats.

#### 4.4.8 Phase Change Property

DSC analysis was conducted using a TA Instruments Discovery DSC 250 Auto under a nitrogen atmosphere. The samples were heated from 0°C to 120°C, with two heating/cooling cycles performed to assess phase change properties. Additionally, 100 cycles were conducted to evaluate the thermal durability of the PCM mats, allowing for the assessment of the material's stability and repeatability during thermal transitions. The DSC curves were analyzed to determine the melting and crystallization temperatures, as well as the latent heat of fusion. The DSC analysis was also performed after the leakage test to investigate any potential loss of PCM.

#### 4.4.9 Photothermal Property

The photothermal response was evaluated by exposing the mats to a simulated solar light source (1.5 sun) and measuring the surface temperature change using an infrared (IR) thermal camera. The experimental setup is shown in **Figure S2**. To evaluate the energy conversion capability, the photothermal conversion efficiency (*η*) of the composite PCMs was determined using the following equation.

(1) $$\text{η}=\frac{m\times \Delta H}{P\times S\times \Delta t}\times 100$$

Here, *m* (g) is the mass of the sample, Δ*H* (J/g) represents the phase change enthalpy, (W/m^2^) is the light intensity, *S* (0.0004909 m^2^) denotes the irradiated area of the sample, and Δ*t* (s) is the duration of the phase transition.

### 4.5 Statistical Analysis

All experiments were conducted in at least three independent replicates, with certain experiments repeated up to five times (*n* = 3–5). Data are reported as mean ± standard deviation (SD). Significant differences between groups were evaluated using one‐way analysis of variance (ANOVA), followed by Tukey's post hoc test, with *p* < 0.05 considered statistically significant. All statistical analyses were performed using OriginPro 2026.

## Supporting Information

Additional supporting information can be found online in the Supporting Information section.

## Author Contributions

**Hossein Baniasadi**: conceptualization (lead), formal analysis (lead), investigation (lead), methodology (lead), project administration (lead), supervision (lead), visualization (lead), writing – original draft (lead), writing – review & editing (lead). **Sedigheh Borandeh**: data curation (equal), formal analysis (equal), methodology (equal). **Ziba Fathi**: conceptualization (equal), methodology (equal), writing – original draft (supporting). **Roozbeh Abidnejad**: formal analysis (equal), investigation (equal), methodology (equal), visualization (equal), writing – original draft (supporting). **Pedro E.S. Silva**: data curation (equal), formal analysis (equal), validation (equal), visualization (equal). **Lauri Välinen**: formal analysis (equal), investigation (equal). **Jaana Vapaavuori**: funding acquisition (lead), writing – review & editing (lead). **Eero Kontturi**: funding acquisition (lead), writing – review & editing (equal). **Jukka Niskanen**: funding acquisition (lead), writing – original draft (equal), writing review & editing (equal).

## Funding

This work was supported by the Academy of Finland (3D‐GRINO‐PPCI); Business Finland (IMD1/Kontturi); Puunjalostusinsinöörit ry.

## Conflicts of Interest

The authors declare no conflicts of interest.

## Supporting information

Supplementary Material

## Data Availability

The data that support the findings of this study are available from the corresponding author upon reasonable request.
