# Supplementary material for: Multifunctional Electrospun Phase Change Material Mats for Solar–Thermal Energy Storage and Photothermal Conversion
Source: Small Sci. 2026 Apr 17;6(4):e202500600. doi: 10.1002/smsc.202500600 (PMC13089636; doi:10.1002/smsc.202500600)
Supplement: Supplementary file 1 — Supplementary Material [file SMSC-6-e202500600-s001.zip › Supplementary information.pdf]

Multifunctional Electrospun Phase Change Material Mats for Solar–Thermal Energy Storage and Photothermal Conversion

Hossein Baniasadi[a]*, Sedigheh Borandeh[a], Ziba Fathi[b], Roozbeh Abidnejad[b], Pedro E.S. Silva[c], Lauri Välinen[a], Jaana Vapaavuori[c], Eero Kontturi[b], Jukka Niskanen[a]

[a]Department of Chemical and Metallurgical Engineering, School of Chemical Engineering, Aalto University, Espoo, Finland
[b]Department of Bioproducts and Biosystems, School of Chemical Engineering, Aalto University, Espoo, Finland
[c]Department of Chemistry and Materials Science, School of Chemical Engineering, Aalto University, Espoo, Finland

* Corresponding author: hossein.baniasadi@aalto.fi

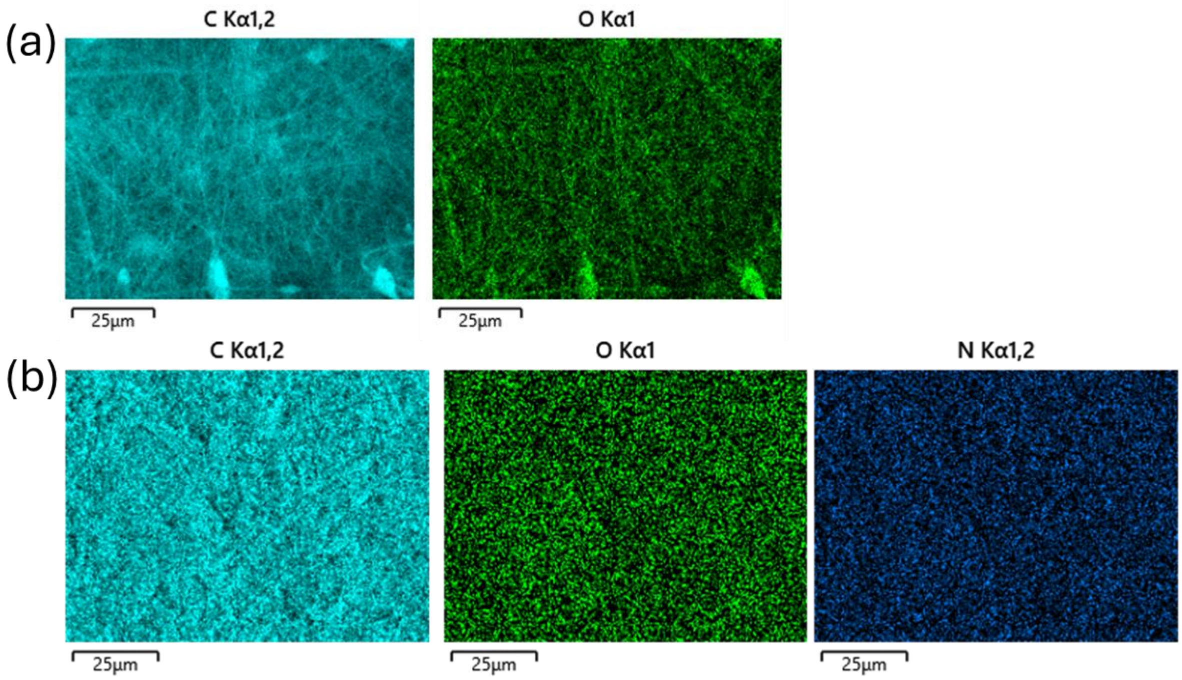

Figure S1. EDX elemental mapping of (a) ePPCM85 fibers showing carbon (C) and oxygen (O) distribution, and (b) PPy@ePPCM85 fibers showing the appearance of nitrogen (N) after polypyrrole polymerization.

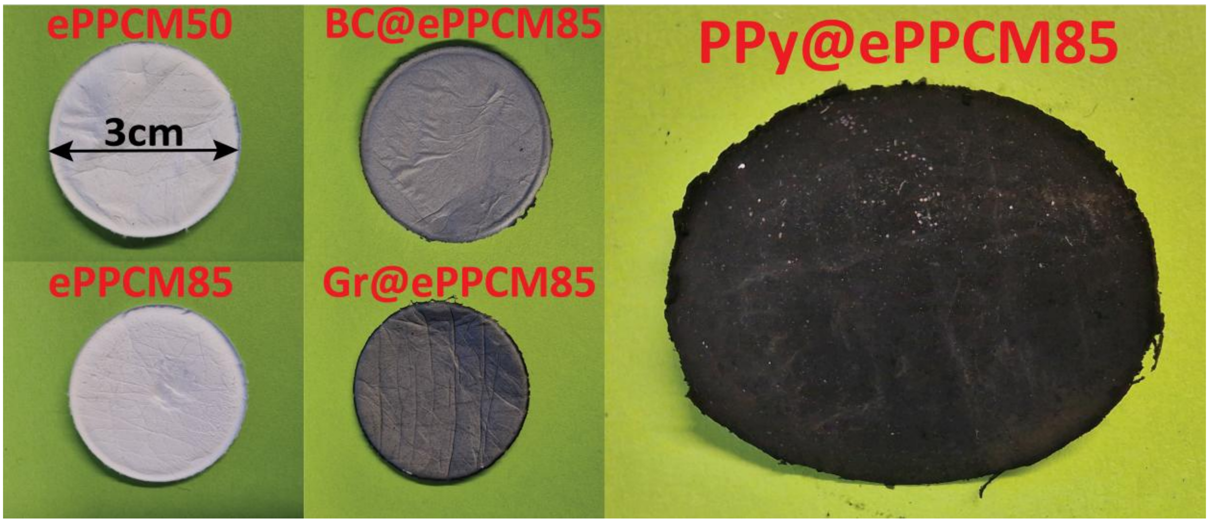

Figure S2. Digital photographs of all mats

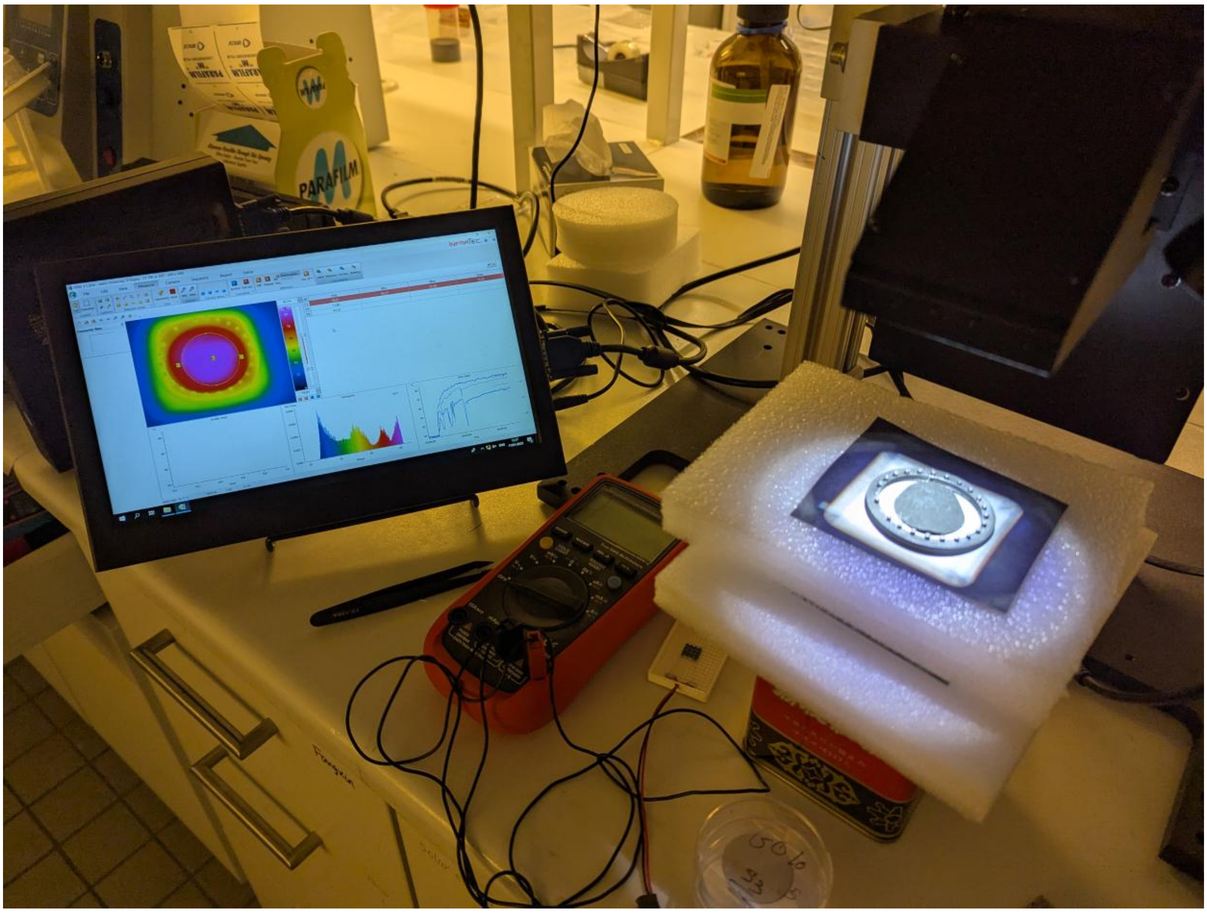

Figure S3. The experimental setup used for photothermal test

Video S1. Flexibility test of PPy@ePPCM85 electrospun mat, illustrating its ability to withstand repeated bending and handling without visible damage.

Video S2. Thermal camera recording of electrospun mats under 1.5 sun irradiation for 300 s, followed by a 300 s cooling period after the light was switched off, demonstrating the photothermal heating and cooling behavior of the samples.
